# Presenting information on dental risk: PREFER study protocol for a randomised controlled trial involving patients receiving a dental check-up

**DOI:** 10.1016/j.conctc.2018.05.009

**Published:** 2018-05-07

**Authors:** Rebecca Harris, Christopher Vernazza, Louise Laverty, Victoria Lowers, Stephen Brown, Girvan Burnside, Laura Ternent, Susan Higham, Jimmy Steele

**Affiliations:** a Department of Health Services Research, Institute of Psychology, Health and Society, University of Liverpool, Liverpool, Merseyside, UK; b School of Dental Sciences, Newcastle University, Newcastle-upon-Tyne, Tyne and Wear, UK; c Department of Psychological Sciences, Institute of Psychology, Health and Society, University of Liverpool, Liverpool, Merseyside, UK; d Department of Biostatistics, Institute of Translational Medicine, University of Liverpool, Liverpool, Merseyside, UK; e Institute of Health and Society, Newcastle University, Newcastle-upon-Tyne, Tyne and Wear, UK

**Keywords:** Oral health, Health education, Risk, Clinical communication, Behaviour change

## Abstract

**Introduction:**

A new dental contract being tested in England places patients into traffic light categories according to risk (Red = High risk). This reflects health policy which emphasises patients' shared responsibility for their health, and a growing expectation that clinicians discuss health risk in consultations. Alongside this, there are technological developments such as scans and photographs which have generated new, vivid imagery which may be used to communicate risk information to patients. However, there is little evidence as to whether the form in which risk information is given is important.

**Methods:**

The PREFER study is a pragmatic, multi-centre, three-arm, patient-level randomised controlled trial, based in four NHS dental practices, from which 400 high/medium risk patients will be recruited. The study compares three ways of communicating risk information at dental check-ups: 1) verbal only (usual care); 2) a Traffic Light graphic with verbal explanation; 3) a Quantitative Light-Induced Fluorescence (QLF) photograph showing, for example, patches of red fluorescence where dental plaque has been present for two days or more (with a verbal explanation). The study assesses patient preferences using the economic preference-based valuation methodology Willingness-to-Pay (WTP). Any changes in oral self-care (for example in tooth-brushing), will be measured by self-report, and clinical outcome data collected by clinicians and extracted from QLF photographs. Predictors and moderators of any behaviour change will be explored using demographic characteristics and psychological variables from the Extended Parallel Process Model. A cost-benefit framework will explore the financial implications for NHS dentistry of the three risk presentation methods.

## 1 Background

The communication of risk information is a fundamental part of nearly all health promotion interventions [1]; and the emphasis on this growing, given government values of freedom, fairness and responsibility articulated in recent health policy [2]. This is reflected in the NHS general dental practice context, where a new model of remuneration is being piloted, based on a care pathway approach which separates patients into ‘Red’ (high), ‘Amber’ (medium), and ‘Green’ (low) risk categories (RAG) [3]. The categorisation is intended to inform conversations about patient self-care behaviours such as eating less sugar and improving tooth-brushing, which are key lifestyle changes known to improve oral health [4].

However, although a link between clinician-patient communication and post-consultation outcomes has been established, the relationship is not straight forward, since relationships between communication behaviour, meaning and evaluation are complex [5]. Communicating disease risk is especially complex, given that risk judgements are ‘imbued by emotion’, and ‘always interpreted via a social and cultural lens’ [6]. Specifically, it is clear that patients do not think about risk as it objectively exists, as a continuum represented by numeric estimates [7,8]. Instead, patients use heuristics, simplified ‘rules of thumb, that allow them to understand and make decisions [[9], [10], [11], [12]]. Thus, the *form* in which risk information is presented to patients is especially important. Providing personalised information in a simplified and accessible way, such as the proposed RAG categories, therefore potentially influences patients’ responses to information on risk. However, little previous research has been undertaken on whether the *form* in which risk information is presented, matters [13]. In particular, no previous studies have compared patients' preferences for different forms of risk information given in a clinical setting [13].

Developments in medical technology means that the range of possible forms in which risk information can be presented to patients has grown - with routine scans and radiographs now able to demonstrate body fat, heart function, osteo-arthritis of joints etc. Previous studies have shown that medical imagery giving a vivid representation of the consequences of unhealthy behaviour can enhance risk communication, although these have used generalised, not personal images, which provide less tailored information about risk status [[14], [15], [16]].

Quantitative Light-Induced fluorescence (QLF) is a recent technological development in the dental field. A QLF camera produces images of teeth, which allows visualisation of tooth mineral loss at a stage before it is visible with the naked eye. It also highlights plaque which has been present in the mouth for more than 48 h [17]. By imaging previously unseen consequences of poor dental self-care, QLF has considerable potential as a risk communication tool, but is, as yet untested.

This study aims to investigate the benefits of two alternative means of communicating risk information to patients: a colour-coded RAG graphic, and a QLF image of their teeth and gums, in support of the usual verbal communication between dentist and patient - comparing these to usual care. Of particular interest, is the value which patients attach to different information forms tested, as measured by Willingness-to-Pay (WTP) – a measure which is widely used in health economics for measuring patients' preferences and determining the economic value of various services [18].

## 2 Methods

### 2.1 Study design

Using a randomised controlled trial design, we will compare patients' valuation and responses to information given 1) verbally (usual care), [V]; 2) verbally accompanied by a traffic light graphic, [TL]; and 3) verbally accompanied by a QLF image, [QLF]. We expect patients to prefer risk information presented in traffic light and/or QLF groups more than usual verbal communication. We also expect to see a greater improvement in oral health behaviours in the traffic light and/or QLF group compared to the usual care group.

### 2.2 Theoretical model

**Fig. 1:**
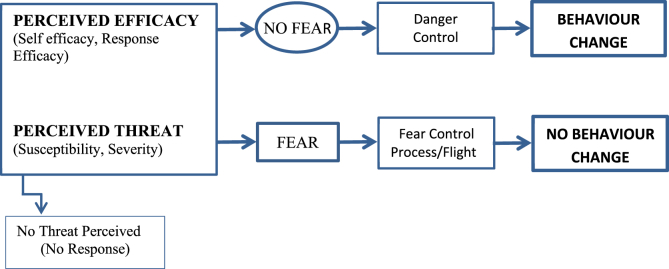
Extended parallel process model.

Imagery and numeric risk estimates are thought to influence people's reaction to risk messages by increasing patients' perception of the said threat to their health and well-being, thus heightening fear regarding any negative consequences of inaction [19]. The Extended Parallel Process Model (EPPM) describes how two appraisals determine whether a risk communication will prompt patients to adopt healthier behaviour (Fig. 1) [20]. Firstly, threat appraisals, (encompassing perceptions that negative health outcomes are likely and severe); are postulated to lead to protective behaviour provided that the coping appraisal is also high. Coping appraisal refers to patients' perceptions that they can change unhealthy behaviour (self-efficacy) [21], and that these changes will reduce risk (outcome efficacy). If coping appraisals are high, generating perceptions of threat and fear are thought to promote behavioural change. On the other hand, if coping appraisals are low, this is thought to lead to defensive behaviours (such as denial of the message), even where individuals perceive themselves to be at risk of a threat [20,22]. Imagery, in particular has been associated with defensiveness [23]. The EPPM points to the possibility that certain risk communications can have negative as well as positive effects on individuals [22]. We will therefore use the EPPM as a framework to help understand why traffic light or QLF supplements to usual verbal risk communication at dental check-ups are or are not effective, and how effectiveness of risk communication may be improved.

### 2.3 Study objectives

• To measure individuals' preferences for three different risk communication forms using Willingness-to-Pay methods.
• To identify any differences in preference for information form between differing demographic, behavioural and psychographic groups.
• To use variables derived from the EPPM model to predict the likelihood that different information leads to behaviour change; and to measure any actual behaviour change, exploring links between behaviour and patients' valuations.
• To conduct a cost-benefit framework analysis of the three different methods and to explore the financial implications for NHS dentistry.

### 2.4 Setting

Patients will be recruited from four NHS dental practices in two areas of the North of England, which are not involved in piloting of the new NHS dental contract using a RAG categorisation for all patients [24]. Practices will be invited to participate by working down a list of randomly numbered NHS dental practices, until two practices in each geographical area are recruited (excluding single-handed practices in view of these being unlikely to generate sufficient patient throughput to meet recruitment targets). Practices expressing an interest in participation will be provided with an information sheet and will consent to take part in the study by the practice owner/s signing a dental practice consent form.

### 2.5 Participants

Participants will be recruited by trained staff at each dental practice. Patients will be approached to take part when making a dental check-up appointment.

#### 2.5.1 Inclusion criteria

**Fig. 2:**
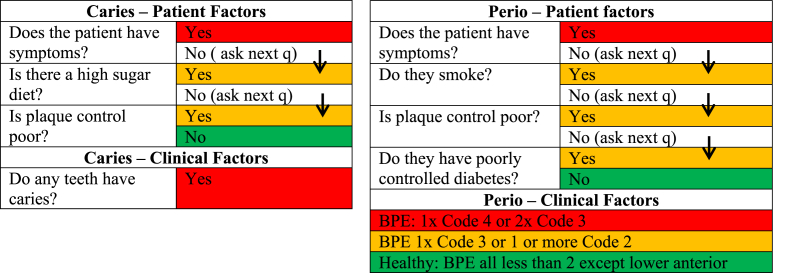
Traffic light algorithm applied to assess patient eligibility (Red/Amber risk patients included). (For interpretation of the references to colour in this figure legend, the reader is referred to the Web version of this article.)

NHS adult patients (aged 18 years or older) deemed to be high/medium risk for poor oral health identified using a nationally developed algorithm, applied by the dental practice [25]. These may be either new patients or regular attenders at that practice. Patients will be screened for eligibility when making the appointment (for example: patient reported symptoms, medical history such as poorly controlled diabetes, and/or health behaviours such as smoking), although eligibility will be fully determined after a clinical examination by a dentist during the dental check-up. This follows the model currently being tested in NHS dental practices where patients are stratified into high/medium risk groups based on a combination of social history/medical history (patient factors) and clinical assessment criteria [26]. For simplicity, clinical criteria for risk assessment are limited to the most common/serious clinical criteria of dental caries and periodontal (gum) disease factors, with soft tissue lesions and non-carious tooth surface loss (erosion, attrition & abrasion) assessment criteria not included (Fig. 2). Risk for caries and periodontal disease are assessed separately, and then the highest risk rating applied to the patient.

#### 2.5.2 Exclusion criteria

Patients identified as ‘Green’ (low risk), based on the absence of either clinical or patient-related factors that increase risk [26] are excluded. Also excluded are patients attending as a new patient for an emergency appointment since they do not routinely receive a full dental check-up; and edentulous patients. Although patients with low literacy will be included in the study, patients who require an interpreter to participate in treatment will be excluded.

### 2.6 Trial interventions

#### 2.6.1 Group 1: Verbal information only (usual care)

**Fig. 3:**
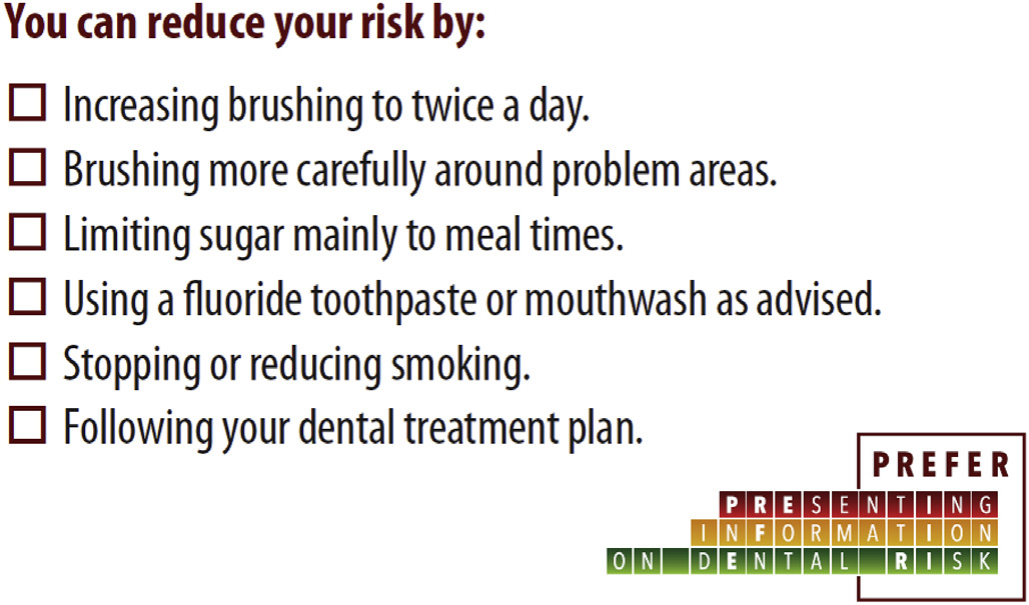
Card showing oral health advice given – any messages given at the check-up being marked by the dentist.

Patients will receive their usual verbal information from the dentist following their check-up. The dentist will mark any of the six main areas of recommended actions covered in the conversation, on a printed credit-card sized card, which will then be given to patient to take away (Fig. 3). The advice ‘*Following your dental treatment plan*’ relates to returning for future dental visits which have been scheduled for that course of treatment.

#### 2.6.2 Group 2: Verbal information plus traffic light graphic

**Fig. 4:**
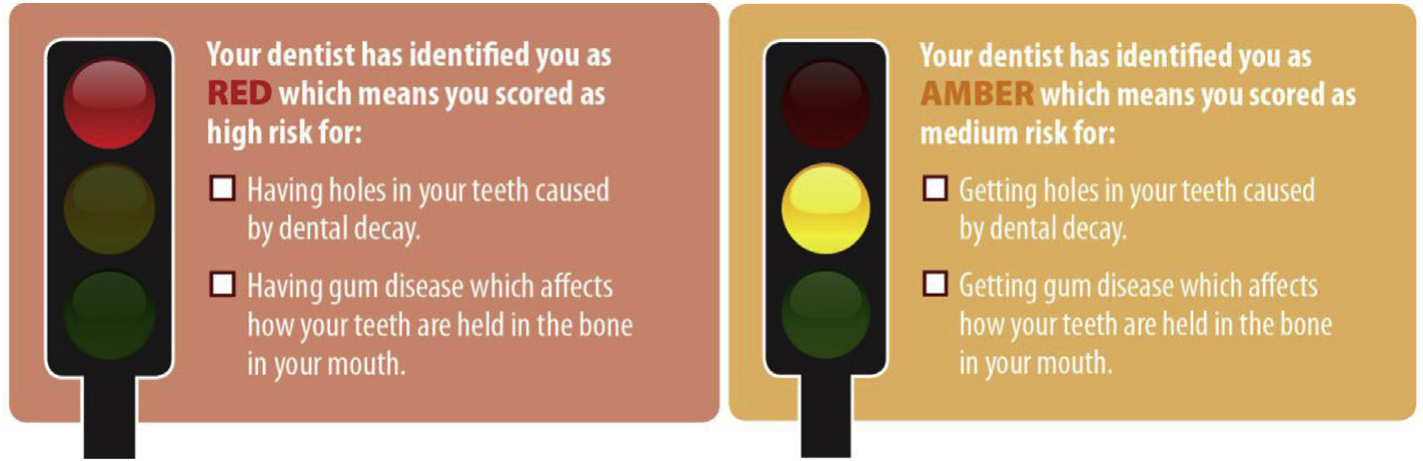
Traffic light graphics on cards given to participants allocated to Group 2 (Traffic Light information).

Patients will receive the same card as Group 1 with any messages covered, marked by the dentist (Fig. 3), but in addition on the reverse side will be a Red/Amber traffic light graphic, corresponding to the risk category to which the patient has been assigned after the clinical assessment (Fig. 4).

#### 2.6.3 Group 3: Verbal information plus QLF photograph

**Fig. 5:**
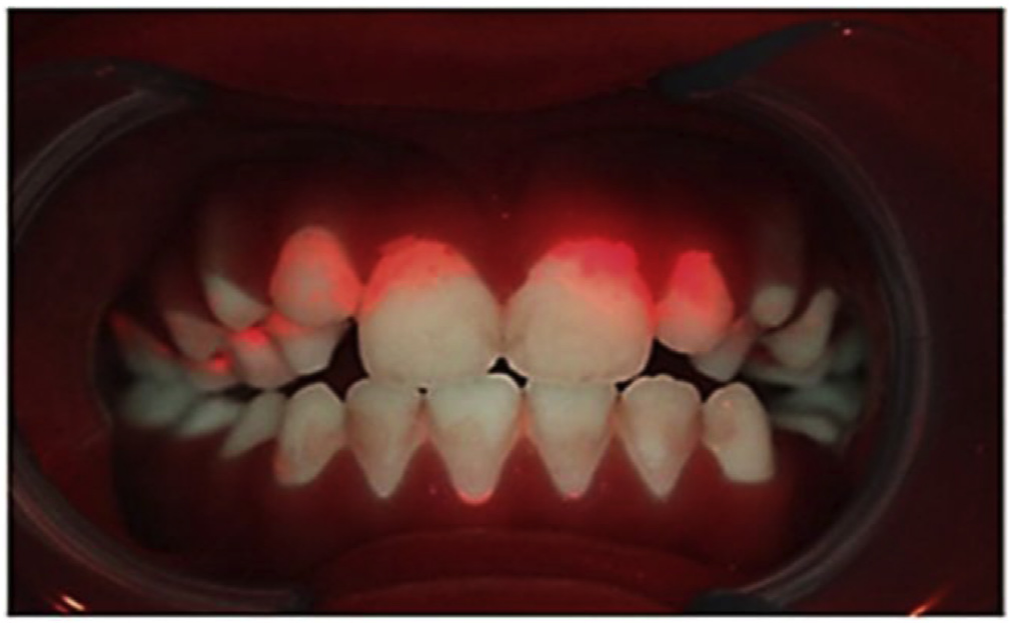
Example QLF photographic image of plaque coverage: Red fluorescence highlighting plaque present in the mouth for over 48 h. (For interpretation of the references to colour in this figure legend, the reader is referred to the Web version of this article.)

The dentist will explain the QLF images (anterior teeth and gums only) to the patient and use this to deliver any preventive advice (Fig. 5). A credit card sized colour copy of the QLF photograph which is most relevant to the advice given (plaque coverage or demineralised areas) will be printed. On the reverse of this card, a sticker will be applied to replicate the Group 1 card (Fig. 3), with any messages covered, marked up by the dentist in the same way.

### 2.7 Randomisation and allocation concealment

The trial will involve simple randomisation of patients into the three arms in a 1:1:1 ratio. Randomisation will occur just after enrolment by dental staff taking a sequentially numbered envelope. The allocation will be revealed by opening the envelope just prior to the information being given (i.e. by the dentist, witnessed by the dental nurse in the dental surgery, after the check-up has been carried out, including baseline clinical outcome assessment – BPE). The allocation sequence will be drawn up by the trial statistician using computer-generated random numbers with block stratification by each of the four dental practices and random variable block sizes. The trial statistician will be blinded to allocation until the final statistical plan is agreed. The researcher extracting clinical outcome data from QLF photographs (plaque coverage and tooth demineralisation) will also be blind to group allocation, as will the researcher gathering 6 and 12 month follow up data.

### 2.8 Outcome measures

#### 2.8.1 Primary outcome (Willingness-to-pay)

Willingness-to-Pay (WTP) will be used to quantify people's preferences for the three forms of information (V, TL, QLF). WTP is recognised as representative of how consumers respond to health care decision making [27]. Other economic preference based measures such as health state utility measures are deemed as unlikely to be sensitive enough to detect small changes in utility; whereas discrete choice experimentation to determine WTP indirectly, are deemed over-complicated for implementation in research based in dental practices [28], which leaves WTP as the most appropriate valuation tool for this context [29].

#### 2.8.2 Secondary outcomes

*Patient-Clinician communication measured immediately after the intervention*

• Communication Assessment Tool (CAT) [30] 15 item stem from *1*=*very poor to 5*=*excellent*

*Self-reported behaviour change between baseline and 6 months post-intervention; and between baseline and 12-months post-intervention*

• Oral hygiene
  o Tooth-brushing frequency *‘How often do you brush your teeth nowadays?’ (1* = >*2x/day, 2*=*Twice a day, 3*=*Once a day, 4* = <*once a day, 5*=*Never)* [31]
  o Duration of tooth-brushing *‘How long do you clean your teeth for nowadays? (1*=>*3 min, 2* = *3 min, 3* = *2 min, 4* = *1 min, 5* = < *1 min)* [32]
  o Frequency of interdental cleaning *‘How often do you do interdental cleaning?* e.g. *with floss, interdental brushes, tooth sticks etc.?’ 1*= > *once a day, 2*=*At least twice a week, 3*=*Weekly, 4*=*Monthly, 5*=*Never* [32]
• Use of Fluoride
  o Fluoride toothpaste (prescribed)
  o Fluoride mouth-rinse
• Dietary sugar
  o Frequency eating/drinking 6 items *‘How often, on average do you eat or drink these things?’ 1*=*More than once a day to 7*=*Never* [33]
  o Frequency sugar in tea and coffee *‘Do you usually have sugar (not artificial sweetener) in hot drinks like tea and coffee?’* [32]
• Smoking [34].
  o Current smoker excluding e cigarettes
  o Average number of tobacco items smoked

*Clinical outcomes*

• Change in self-rated oral health between baseline and 6 months post-intervention ‘*Would you say your dental health (mouth, teeth and/or dentures) is? 1*=*very good to 5* = *very poor* [31]
• Basic Periodontal Examination (BPE) - clinical data collected by the dentist during the check-up: conversions between codes 1 (bleeding) and 0 (health) between baseline and next dental visit
• Change in plaque coverage: percentage of the area of buccal/labial surfaces of anterior teeth calculated from QLF images (Plaque Percentage Index (PPI), ΔR30) [35] taken at baseline and next dental visit. The QLF anterior teeth image will involve a photograph of maxillary and mandibular teeth from canine to canine taken without overlap of incisal edges.
• Number of tooth surfaces affected by early caries: Change between baseline and the next dental visit in the number of surfaces which are demineralised on the buccal/labial surfaces of anterior teeth as a proportion of number of surfaces available [35]. Teeth will be cleaned (brushed by the participant after taking the QLF image to measure plaque), before a second QLF image measuring caries.
• ΔQ: Change between baseline and the next dental visit in percentage fluorescence loss based on the fluorescence of sound tissue multiplied by the area. This is an estimate of lesion volume, and can be combined over all lesions to give a total estimate of overall severity per patient [35]. Calculated from QLF image taken after cleaning.

### 2.9 Predictor and moderator variables

*Patient socio-demographic characteristics*
  • Area level deprivation based on home postcode (IMD) [36].
  • Employment status [36] response from *1*=*employed/self-employed full-time, 2*=*employed or self-employed part-time, 3*=*unemployed at the moment, 4* = *full-time education*
  • Household income (before deductions e.g. income tax) [37] *according to 9 categories given as weekly or yearly amounts, ranging from 1* = *£0 to 9* = *over £52,000* per *year*
  • Highest level of education response from *1* = *GCSE/NVQ level 1 or similar, to 5* = *postgraduate degree* [37].
  • Literacy using the Rapid Estimate of Adult Literacy in Medicine (REALM) [38]. The 8 medical test words, as well as the 3 practice words will be printed and shown to participants on an A4 laminated sheet, but with the American spelling of anemia changed to the English version (anaemia).
*Patient dental visiting behaviour, dental anxiety and previous dental experiences*
  • Dental visiting: *‘In general, do you go to the dentist for: 1*=*a regular check-up, 2*=*an occasional check-up, 3*=*/only when having trouble with my teeth/dentures?’* [31]. *‘How many times have you been to the dentist in the last five years purely for a check-up?’* [31]
  • Previous pain experience: *‘Have you ever experienced dental pain bad enough to make you go to the dentist (tick all that apply): 1*=*currently in pain, 2*=*In the last 6 months, 3*=*6 months to 2 years ago, 4*=*more than 2 years ago, 5*=*never)’* [31].
  • Previous experience of dental treatment: *‘Have you ever had fillings/teeth extracted (taken out)/a dental bridge or a tooth crowned/a root canal treatment/a scale and polish?’* [31]
  • Dental anxiety: The Modified Dental Anxiety Scale (MDAS) [39].
*Dental provider characteristics*
  • Dental practice
  • Clinician ID delivering information
*Oral health*
  • Number of natural teeth as recorded by the dentist at the check-up
*Risk perception and behaviour change predictors/moderators (based on the EPPM model)* [20]
  • Perceived threat (severity) *‘How serious would it be to you if* [negative outcome] *were to occur,* response from *1*=*not at all serious to 5*=*absolutely serious* Five possible negative outcomes of poor oral health were identified in earlier qualitative work: *‘your teeth were to make you feel uncomfortable when smiling, talking and laughing in front of people; people thought you had failed to look after your own teeth; your teeth were to become more painful and sensitive; you were to need treatment which meant spending more time at the dentist; you were to need treatment which you could not afford’*
  • Perceived threat (susceptibility) ‘*If you do not follow the dentist's advice, how likely is it that* [same 5 negative outcomes] *will occur*’ response from *1*=*absolutely unlikely to 5 absolutely likely*
  • Self-efficacy ‘*Please consider how confident you are that you can perform the behaviour properly, regularly and on a long-term basis for* [target behaviour]’ *response* from *1*=*not at all confident to 5*=*absolutely confident*.
  • Response efficacy ‘[target behaviour] *means I am less likely to have dental problems*’ responses from *1*=*Absolutely disagree to 5*=*Absolutely agree*
  • Message Fear ‘*How much did the advice you received make you feel … frightened/tense/nervous/anxious/uncomfortable*’ response from *1*=*not at all to 5*=*very much*
  • Affect regarding threat ‘*How would you feel how if you experienced* [negative outcome] [40]; *and* ‘*How vulnerable would you feel if you experienced* [negative outcome] [41] responses from *1*=*not bad to 5*=*absolutely bad*
  • Danger control response measured using 3 items: ‘*How likely it will be before your next appointment that you will. 1) follow the advice given by the dentist completely; 2) follow some of the advice given by the dentist 3) talk to someone about the advice the dentist gave you*’ response from *1*=*absolutely unlikely to 5*=*absolutely likely*
  • Fear control response measured using 4 items with stems: 1) Defensive Avoidance: ‘*I prefer not to think about the advice given to me by the dentist*’ 2) Perceived Manipulation: ‘*The advice given to me by the dentist is untrue or manipulated*’ and 3/4) Message Derogation: ‘*The advice given to me by the dentist is exaggerated*’ and ‘*I do not personally believe the advice given to me by the dentist*’ responses from *1*=*strongly disagree to 5*=*strongly agree*
  • Intention to change behaviour ‘*Before my next appointment I intend to*.[target behaviour]’ responses from *1* = *absolutely disagree to 5* = *absolutely agree*.

### 2.10 Sample size calculation

The required sample size calculation is based on a need to detect significant differences in the primary outcome (WTP) between the two interventions (Traffic Light and QLF) and usual care arms at 80% power with α = 0.05. Sample sizes for WTP studies are recognised as difficult, given the problems in deciding on a minimally important difference amongst others [42]. Moreover, for this study, no previous WTP valuations have been undertaken for similar “goods” i.e. information based “goods” in health. In the absence of reliable standard deviation and effect size estimates, sample size was calculated with effect sizes based on numbers of standard deviations rather than absolute numbers. Thus, to detect a difference of 0.5 standard deviations 63 people is calculated to be required per arm. To detect 0.33 standard deviations, 145 per arm would be needed. Accepting a detectable difference between half and a third of a standard deviation and allowing for around 20% refusal to answer WTP questions (protest responses) gives a figure of 133 in each arm or a total sample size of 400. We then calculated the implication of this sample size for the detection of clinical outcome effects, and based this on the secondary outcome of PPI, measured using QLF [35]. Published data on a group of 38 college students showed a mean PPI of 14.8, with a standard deviation of 7.7 [43]. As this is likely to be a more homogenous population than our study, a more conservative estimate of standard deviation of 10 has been used to calculate a sample size of 133 per group would allow us to detect a mean different of 3.5 in PPI between groups, with 80% power, at the 0.05 significance level.

### 2.11 Trial process and trial-specific training

All practices will receive two separate sets of training. First in relation to the conduct of the trial – the whole dental team including the receptionist, will receive training in Good Clinical Practice and trial procedures (e.g. patient consent, randomisation procedures and completing study records). A crib sheet detailing study procedures in all three arms will be provided for reference. Secondly, researchers will train dental teams in trial-specific processes including how to take (whole dental team) and clinically interpret (dentists only) QLF photographs, again supported by written information, and a video about the use of the camera. Both types of training will be undertaken in the dental practice itself. Given that research identifies that verbal metaphors can influence patients' mental images of their condition [44], trial specific training will include standard messages such as ‘*This red patch on the QLF photograph shows bacteria which have not been cleaned by you for 2–3 days. If you do not improve tooth-brushing here you are highly likely to develop problems’*.

### 2.12 Data collection procedures

**Fig. 6:**
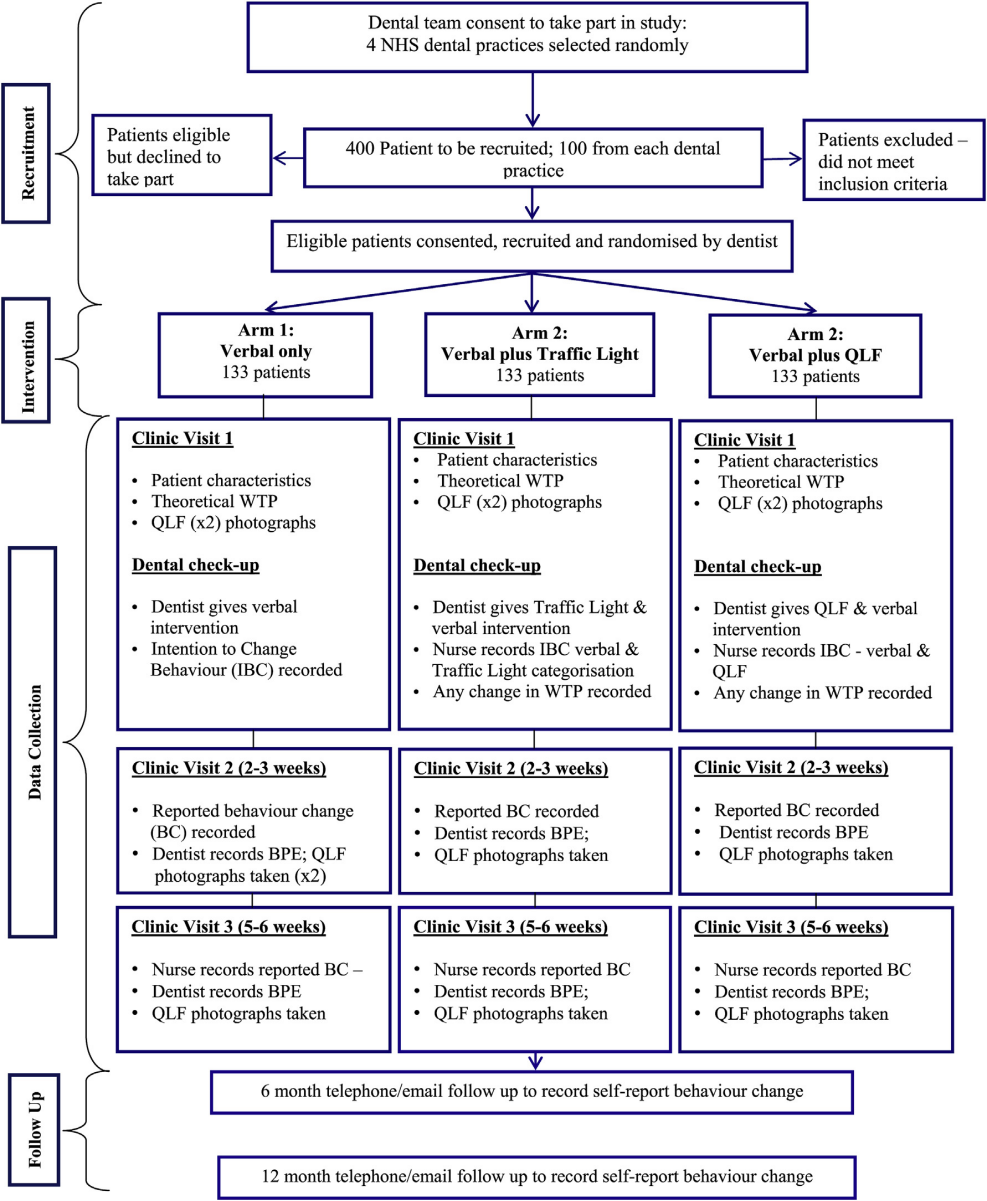
Study flow diagram.

Data will be collected at baseline, then 2–3 weeks later when the patient returns for their first treatment visit (second dental visit, V2), and then at 5–6 weeks post intervention (third dental visit, V3), (Fig. 6, Table 1). Since eligible patients are those at medium/high risk of poor oral health, we expect the patient to need to return for further care, and so we will collect data opportunistically at this time. At these V2 and V3 follow up visits, the dentist will measure BPE (before the patient receives usual care treatment), and a further QLF photograph will be taken (not shown to the patient) to enable a change in plaque coverage and demineralisation to be measured from QLF images. Participants will be asked about any change in behaviour in relation to the messages (Fig. 3) listed on the printed cards (given to all patients at the first visit), using the item stem ‘*Since my last appointment I have* …. ’.

**Table 1:** Data collection schedule.

TIME POINTS	Baseline (Visit 1)	Visit 2	Visit 3	6-months	12-months
Collection method					
**Assessments – self report**					
Patient demographics	x				
REALM-R	x				
WTP	x				
Dental visiting history/experience	x				
Dental anxiety (MDAS)	x				
Self-assessed oral health	x	x	x	x	x
Oral health behaviours: toothbrushing, diet, fluoride and smoking	x	x	x	x	x
EPPM and behaviour change predictors	x	x		x	x
Patient/clinician communication (CAT)	x				
**Assessments – clinical**					
QLF photographs	x	x	x		
Number of natural teeth	x				
BPE	x	x	x		

There will be two further follow up points at 6 and 12 months post intervention, involving questions completed either by telephone or email, depending on what contact information the patient gave for this purpose. For those who cannot be contacted using this method, postal questionnaires will be sent. This will be conducted by a trained member of the research team. Patients who complete follow-up will be given the opportunity of being entered into a prize draw.

Measures will be collected according to the schedule outlined in Table 1. The patients will enter some data themselves using a Tablet computer with Qualtrics survey software (Version 092,017, ^©^ 2017 Qualtrics^®^). Trained dental nurses will administer an assessment of literacy (REALM), enter some demographic details and take QLF photographs.

#### 2.12.1 Measuring WTP

This will be self-completed by patients on the Tablet PC platform, supported by a trained dental nurse, where required. The WTP elicitation will occur before the patient has been given risk information in any form; so all participants will give values for all three interventions, regardless of subsequent randomisation to one intervention. Participants will be given descriptions and sample images of the three interventions (Fig. 3, Fig. 4, Fig. 5), and asked to rank them in order of preference, and then asked firstly for their WTP for the lowest ranked intervention. This will be supported by a script which encourages realistic, budget constrained responses and which emphasises that the exercise is about value rather than price.

On the computer participants will be presented with a series of virtual cards with different values from 50 pence to £150 on them and asked to drag each card to one of three boxes: “*Would pay; Wouldn't pay; Not sure”.* The different valued cards will be presented in a random order, with a random starting card. Participants will not see the value of subsequent cards until the current card has been placed. Once all cards have been placed, the lowest “*Wouldn't pay*” and highest “*Would pay*” value will be shown, and the participant asked for a maximum WTP value in an open-ended format. This shuffled payment card approach to WTP elicitation is thought to reduce starting point and range bias found in WTP methods [45].

Once a WTP value for the lowest ranked intervention has been determined, participants will then be asked what extra they would be willing to pay for their next most preferred intervention in an open-ended question. Finally, participants will be asked what extra they would be willing to pay on top of their value for their middle-preferred intervention. This incremental approach to eliciting WTP has been shown to give more robust valuations [46].

After receiving information at their check-up, patients will return to the Tablet PC-based task, where they will be reminded of the WTP value they had given prior to the intervention which had been allocated to receive. They will then be asked if they would revise their WTP, given their actual experience with that form of information. If they indicate they would revise this, the new value will be collected using an open-ended question.

### 2.13 Analysis plan

A detailed statistical analysis plan will be prepared prior to data analysis.

#### 2.13.1 Analysis of WTP

We will compare hypothetical WTP across the whole sample, across the three arms. We will also compare hypothetical WTP with WTP for a good that has been “consumed” by comparing WTP before and after receiving the information form in that arm. WTP data will be analysed both descriptively, comparatively and econometrically. Descriptive data analysis will consist of WTP value means with standard deviations, along with medians and quartiles for each information form. The WTP values for each system will be compared using appropriate comparative tests (e.g. most likely to be Mann Whitney U tests given the likely distribution). In order to understand fully how various dental and demographic factors influence values (WTP), econometric regression analyses will be carried out.

#### 2.13.2 Analysis of behavioural outcomes

Group effects on oral health behaviour between the conditions will be assessed by predicting six and twelve-month follow-up scores from group membership. To minimise the probability of Type 1 error, multivariate analyses will be used. Subsequent univariate analyses will identify specific variables affected by group. Planned comparisons will test the hypotheses that; TL leads to greater positive oral health behaviour change than V, and QLF leads to greater positive behavioural change than V. Separate analyses will examine changes from baseline to 6 months and from baseline to 12 months.

Moderation analyses involve the modelling of two-way interaction effects from the product of the predictor and moderator variables and testing the hypothesis that the interaction term uniquely predicts behavioural change controlling main effects terms [47]. Regression analyses will be used to test this hypothesis. Demographic, dental health and dental visiting variables will be tested as potential moderators.

Mediation analyses assess the extent to which intermediate variables (mediators) explain variance shared between predictor and outcome variables. Again, this analysis will be performed only where between group differences in behavioural change have been identified. Our mediation analysis strategy is based on recommendations by Zhao et al., [48] who specify pre-conditions that the predictor (group membership) be linked to the mediator (EPPM variables), and that the mediator be linked to the criterion (behavioural change) controlling the predictor. When these are established, path analysis can be used to estimate the mediation effect. To statistically test mediation, we will use a bootstrapping method [49]. All potential mediators will be assessed in the same analysis, reducing the likelihood of Type 2 error that would be associated with testing mediators separately.

#### 2.13.3 Analysis of clinical outcomes

Clinical outcomes will be analysed at the first short term follow-up visit, no more than three months post-randomisation. All QLF images will be rated as good/poor quality by the assessor (blinded to group allocation). Those not judged to be of sufficient quality to accurately generate the outcome variables will be excluded from the analysis. Analysis of the three QLF generated variables will use general linear models, with value of the variable of interest at the first short-term follow-up appointment as the outcome variable, and intervention group allocation as a fixed factor. Baseline value of the variable of interest will be included as a covariate. All analyses will follow the intention to treat principle as far as is practically possible.

Basic Periodontal examination (BPE) scores will be analysed by categorising each patient into one of four outcome categories. 1 – stable healthy (code 0 at baseline and follow-up), 2- stable bleeding (code 1 or greater at baseline and follow-up), 3 – change to bleeding (code 0 at baseline, code 1 or greater at follow-up), and 4 – change to healthy (code 1 or greater at baseline, code 0 at follow-up). The analysis will use multinomial logistic regression to test the effect of intervention group on outcome, with the primary hypothesis of interest between groups 2 and 4.

Multiple imputation analyses will be carried out to investigate the robustness of the results to missing data. Using all the data which is available, including baseline variables, five complete case data sets will be imputed, and each analysed in the same way. The results can then be combined, and compared with the analysis excluding missing data, to assess whether this would be likely to change the overall conclusions.

### 2.14 Ethical considerations

Liverpool Health Partners is the research sponsor (Approval reference no: UoL001042). Favourable ethical opinion for the study was confirmed by the North West, Liverpool-East National Research Ethics Committee on 1.8.14 (REC reference number 14/NW/1016). A subsequent substantial amendment in ethical approval was obtained on 18.3.16 to allow a prize draw of ten lots of £25 for patients completing follow up data collection at 6 and again at 12 months (£500 total). NHS Research Governance approval was obtained from The Royal Liverpool and Broadgreen University Hospitals NHS Trust on 20.8.14 (reference number 4819).

Participants meeting eligibility criteria will be given a Patient Information Sheet by dental practice staff which will provide all details of the study procedures, rights to withdrawal, anonymity, confidentiality, along with details of the research team; and have the opportunity to ask questions before informed consent is taken by a member of dental staff trained in GCP.

Participant details will be recorded on a recruitment log to facilitate follow-up data collection. Participants will be assured that all data will be anonymised and stored on a database under the guidelines of the 1998 Data Protection Act. No patient-identifiable information will be sent via electronic means (use of coded study number, patient's sex and patient's age only). Any patient identifiable information (e.g. recruitment logs), will be collected in person from dental practices by a member of the research team rather than electronically. Data and study documentation will be archived at the University of Liverpool for at least five years after the completion of the study in line with associated regulations.

## 3 Discussion

Reform of the NHS dental contract is based on a reorientation from a treatment-focused service, to one which prioritises prevention of disease; with patients participating by improving their oral health behaviours to minimise the (re-)occurrence of disease. The idea follows a general drive towards emphasising that patients have a shared responsibility for maintaining their health. Over 75 NHS dental practices are currently testing two alternative prototypes in England; both using a categorisation of patients according to a Traffic Light scheme, with this information communicated to patients at their dental check-up. This study is therefore set to directly inform national policy, relating to whether patients value risk information in this form. A recent non-randomised study in six NHS dental practices which compared a new model involving a traffic light risk assessment of patients, with other practices using the previous model of dental practice care, found only 291 of 550 participants recruited attended both baseline and follow up appointments (53%). This suggests that long term follow up of patients in a study such as this may present challenges [50]; and recruitment of dental patients to a trial set in dental practice, especially those with a high/medium risk of oral disease, may present further challenges [51]. Nevertheless, when considered against the continuum of pragmatic as opposed to explanatory trials, this study is designed to be at the pragmatic end of the spectrum, since its purpose is to examine the intervention (communication of risk information) under the usual conditions in which they will be applied [52]. The trial will be supported by embedded qualitative work to support understanding the contextual issues influencing the implementation of the intervention, and the trial itself.

### 3.1 Study status

The first patient was recruited in August 2015. Twelve-month follow up data collection will be completed by October 2017.

## Contributors

RH, JS, CV, LT, SB, and GB designed the study. RH, SB, LL, and VL designed self-report measures. LL, RH, CV, SH, contributed to the design of data collection procedures. RH, SB, CV and GB designed the analysis plan. RH drafted the manuscript, with all authors approving the final manuscript. All authors will meet regularly to ensure the smooth running of the study.

## Funding

This study is funded by the National Institute for Health Research (NIHR), Health Services and Delivery Research [HS & DR] programme (project number 13/33/45 PREFER The cost and value of different Forms of information on oral health status and risk given to patients following a check-up in dental practice: A Randomised Controlled Trial). This report is independent research commissioned by NIHR.

## Department of health disclaimer

The views and opinions expressed herein are those of the authors and do not necessarily reflect those of the Health Services and Delivery Research programme, NIHR, NHS or the Department of Health.

## Competing interests

None.

## Provenance and peer review

The study is funded by NIHR through their researcher-led programme (not commissioned). Ethical approval received following peer review.

## Appendix A Supplementary data

Data profile Data profile
